# (2*E*)-1-(3,4-Dichloro­phen­yl)-3-(2-hy­droxy­phen­yl)prop-2-en-1-one

**DOI:** 10.1107/S1600536812000505

**Published:** 2012-01-11

**Authors:** Jerry P. Jasinski, James A. Golen, Prakash S. Nayak, B. Narayana, H. S. Yathirajan

**Affiliations:** a Department of Chemistry, Keene State College, 229 Main Street, Keene, NH 03435-2001, USA; b Department of Studies in Chemistry, Mangalore University, Mangalagangotri 574 199, India; c Department of Studies in Chemistry, University of Mysore, Manasagangotri, Mysore 570 006, India

## Abstract

In the title compound, C_15_H_10_Cl_2_O_2_, the dihedral angle between the mean planes of the two benzene rings is 7.7 (6)°. The crystal packing is influenced by O—H⋯O hydrogen bonds, which form chains along [010]. Weak π–π stacking inter­actions [centroid–centroid distance = 3.6697 (13) Å] are observed, which may contribute to the crystal packing stability.

## Related literature

For the pharmacological activity of chalcones, see: Bandgar *et al.* (2010 ▶); Cheng *et al.* (2008 ▶); Dhar (1981 ▶); Dimmock *et al.* (1999 ▶); Nowakowska (2007 ▶). For the synthesis of chalcone derivatives, see: Samshuddin *et al.* (2010 ▶; 2011 ▶); Fun *et al.* (2010 ▶); Jasinski *et al.* (2010 ▶); Baktır *et al.* (2011 ▶). For related structures, see: Fun *et al.* (2011 ▶); Jasinski *et al.* (2011 ▶).
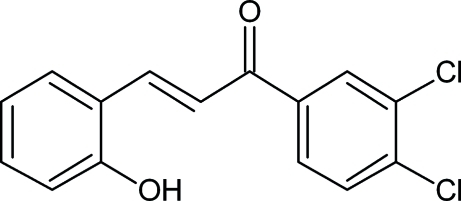


## Experimental

### 

#### Crystal data

C_15_H_10_Cl_2_O_2_
*M* *_r_* = 293.13
Triclinic, 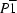

*a* = 7.2551 (6) Å
*b* = 7.8351 (7) Å
*c* = 12.8049 (11) Å
α = 92.367 (7)°
β = 102.946 (8)°
γ = 109.011 (8)°
*V* = 665.51 (10) Å^3^
*Z* = 2
Mo *K*α radiation
μ = 0.48 mm^−1^
*T* = 173 K
0.34 × 0.15 × 0.06 mm

#### Data collection

Oxford Diffraction Xcalibur Eos Gemini diffractometer
Absorption correction: multi-scan (*CrysAlis RED*; Oxford Diffraction, 2010 ▶) *T*_min_ = 0.854, *T*_max_ = 0.972
5430 measured reflections
3174 independent reflections
2417 reflections with *I* > 2σ(*I*)
*R*_int_ = 0.018

#### Refinement

*R*[*F* ^2^ > 2σ(*F* ^2^)] = 0.041
*wR*(*F* ^2^) = 0.115
*S* = 1.01
3174 reflections
175 parameters
1 restraint
H atoms treated by a mixture of independent and constrained refinement
Δρ_max_ = 0.30 e Å^−3^
Δρ_min_ = −0.26 e Å^−3^

### 

Data collection: *CrysAlis PRO* (Oxford Diffraction, 2010 ▶); cell refinement: *CrysAlis PRO*; data reduction: *CrysAlis RED* (Oxford Diffraction, 2010 ▶); program(s) used to solve structure: *SHELXS97* (Sheldrick, 2008 ▶); program(s) used to refine structure: *SHELXL97* (Sheldrick, 2008 ▶); molecular graphics: *SHELXTL* (Sheldrick, 2008 ▶); software used to prepare material for publication: *SHELXTL*.

## Supplementary Material

Crystal structure: contains datablock(s) global, I. DOI: 10.1107/S1600536812000505/vm2148sup1.cif

Structure factors: contains datablock(s) I. DOI: 10.1107/S1600536812000505/vm2148Isup2.hkl

Supplementary material file. DOI: 10.1107/S1600536812000505/vm2148Isup3.cml

Additional supplementary materials:  crystallographic information; 3D view; checkCIF report

## Figures and Tables

**Table 1:** Hydrogen-bond geometry (Å, °)

*D*—H⋯*A*	*D*—H	H⋯*A*	*D*⋯*A*	*D*—H⋯*A*
O2—H2*O*⋯O1^i^	0.84 (2)	1.88 (2)	2.7168 (17)	176 (2)

Symmetry code: (i) 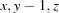.

**Fig. 1.**
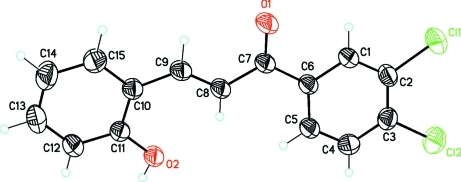
Molecular structure of the title compound showing the atom labeling scheme and 50% probability displacement ellipsoids.

**Fig. 2.**
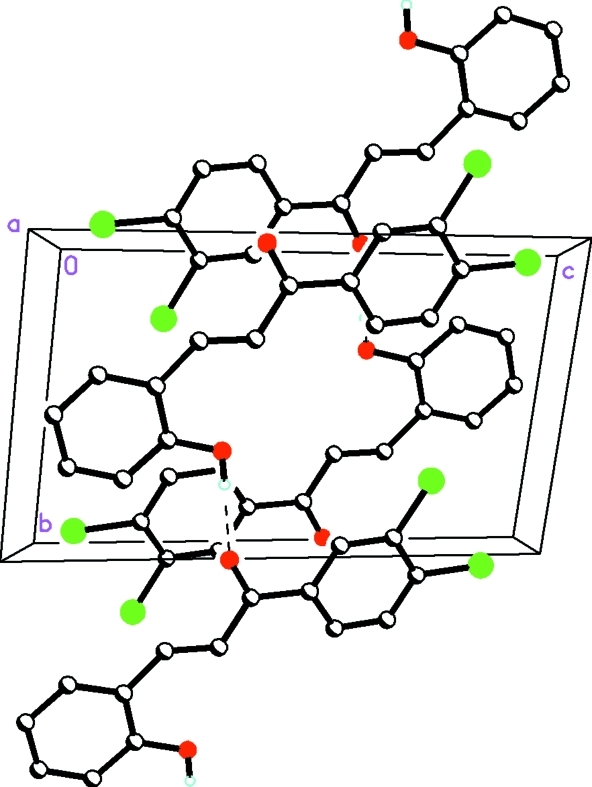
Packing diagram of the title compound viewed along the a axis. Dashed lines indicate O—H···O hydrogen bonding. The remaining hydrogen atoms have been omitted for clarity.

## supplementary crystallographic information

### Comment

Chalcones are abundant in edible plants and considered as the precursors of flavonoids and isoflavonoids. They have also been shown to display a diverse array of pharmacological activities (Dhar, 1981; Nowakowska, 2007) including anti-infective, anti-inflammatory, antimicrobial, antifungal, antioxidant, cytotoxic, antitumor, anticancer and mutagenic properties (Dimmock *et al.*, 1999; Cheng *et al.*, 2008; Bandgar *et al.*, 2010). The basic skeleton of chalcones which possess an α,β-unsaturated carbonyl group is a useful synthone for the synthesis of various biodynamic cyclic derivatives such as pyrazoline, isoxazoline, 2,4,6-triaryl pyridine, benzodiazepine and cyclohexenone derivatives (Samshuddin *et al.*, 2010; 2011; Fun *et al.*, 2010; Jasinski *et al.*, 2010; Baktır *et al.*, 2011). The crystal structures of some chalcones, *viz*., (2E)-3-[3-(benzyloxy)phenyl]-1-(2-hydroxyphenyl)prop-2-en-1-one (Fun *et al.*, 2011), (2E)-3-(4-chlorophenyl)-1-(4-hydroxyphenyl) prop-2-en-1-one (Jasinski *et al.*, 2011), have been reported. In continuation of our studies on chalcones and their derivatives, the title compound (I) was prepared and its crystal structure is reported.

In the title compound, C_15_H_10_Cl_2_O_2_, the dihedral angle between the mean planes of the two benzene rings is 7.7 (6)° (Fig. 1). O—H···O hydrogen bonds (Table 1) are observed between the hydroxyl hydrogen and propene oxygen atoms forming 1-D polymeric chains along [010]. In addition, weak π–π stacking interactions (*Cg*1···*Cg*2 distance of 3.6697 (13) Å; *Cg*1 and *Cg*2 are the centroids of the C1–C5 ring and C10–C15 ring, respectively) are observed which may contribute to crystal packing stability.

### Experimental

To a mixture of 2-hydroxybenzaldehyde (1.22 g, 0.01 mol) and 3,4-dichloroacetophenone (1.89 g, 0.01 mol) in ethanol (40 ml), 10 ml of 10% sodium hydroxide solution was added and stirred at 278–288 K for 3 h. The precipitate formed was collected by filtration and purified by recrystallization from ethanol. Single crystals were grown from DMF by the slow evaporation method. The yield of the compound was 86%. (M.P.: 392 K).

### Refinement

The H2O atom was located by a difference map and refined isotropically with *DFIX* = 0.85 (2) Å. All of the remaining H atoms were placed in their calculated positions and then refined using the riding model with C—H lengths of 0.93 Å. Isotropic displacement parameters for these atoms were set to 1.19–1.20 (CH) times *U*_eq_ of the parent atom.

### Crystal data

**Table:**

C_15_H_10_Cl_2_O_2_	*Z* = 2
*M**_r_* = 293.13	*F*(000) = 300
Triclinic, *P*1	*D*_x_ = 1.463 Mg m^−^^3^
Hall symbol: -P 1	Mo *K*α radiation, λ = 0.71073 Å
*a* = 7.2551 (6) Å	Cell parameters from 1666 reflections
*b* = 7.8351 (7) Å	θ = 3.1–30.0°
*c* = 12.8049 (11) Å	µ = 0.48 mm^−^^1^
α = 92.367 (7)°	*T* = 173 K
β = 102.946 (8)°	Plate, pale yellow
γ = 109.011 (8)°	0.34 × 0.15 × 0.06 mm
*V* = 665.51 (10) Å^3^	

### Data collection

**Table:**

Oxford Diffraction Xcalibur Eos Gemini diffractometer	3174 independent reflections
Radiation source: Enhance (Mo) X-ray Source	2417 reflections with *I* > 2σ(*I*)
graphite	*R*_int_ = 0.018
Detector resolution: 16.1500 pixels mm^-1^	θ_max_ = 27.9°, θ_min_ = 3.3°
ω scans	*h* = −9→9
Absorption correction: multi-scan (*CrysAlis RED*; Oxford Diffraction, 2010)	*k* = −9→10
*T*_min_ = 0.854, *T*_max_ = 0.972	*l* = −16→16
5430 measured reflections	

### Refinement

**Table:**

Refinement on *F*^2^	Primary atom site location: structure-invariant direct methods
Least-squares matrix: full	Secondary atom site location: difference Fourier map
*R*[*F*^2^ > 2σ(*F*^2^)] = 0.041	Hydrogen site location: inferred from neighbouring sites
*wR*(*F*^2^) = 0.115	H atoms treated by a mixture of independent and constrained refinement
*S* = 1.01	*w* = 1/[σ^2^(*F*_o_^2^) + (0.0532*P*)^2^ + 0.1793*P*] where *P* = (*F*_o_^2^ + 2*F*_c_^2^)/3
3174 reflections	(Δ/σ)_max_ < 0.001
175 parameters	Δρ_max_ = 0.30 e Å^−^^3^
1 restraint	Δρ_min_ = −0.26 e Å^−^^3^

### Special details

**Table:**

Geometry. All e.s.d.'s (except the e.s.d. in the dihedral angle between two l.s. planes) are estimated using the full covariance matrix. The cell e.s.d.'s are taken into account individually in the estimation of e.s.d.'s in distances, angles and torsion angles; correlations between e.s.d.'s in cell parameters are only used when they are defined by crystal symmetry. An approximate (isotropic) treatment of cell e.s.d.'s is used for estimating e.s.d.'s involving l.s. planes.
Refinement. Refinement of *F*^2^ against ALL reflections. The weighted *R*-factor *wR* and goodness of fit *S* are based on *F*^2^, conventional *R*-factors *R* are based on *F*, with *F* set to zero for negative *F*^2^. The threshold expression of *F*^2^ > σ(*F*^2^) is used only for calculating *R*-factors(gt) *etc*. and is not relevant to the choice of reflections for refinement. *R*-factors based on *F*^2^ are statistically about twice as large as those based on *F*, and *R*- factors based on ALL data will be even larger.

### Fractional atomic coordinates and isotropic or equivalent isotropic displacement parameters (Å^2^)

**Table:**

	*x*	*y*	*z*	*U*_iso_*/*U*_eq_	
Cl1	0.23632 (10)	1.23387 (8)	0.22164 (5)	0.0692 (2)	
Cl2	0.34479 (10)	0.94069 (9)	0.09406 (4)	0.0681 (2)	
O1	0.2056 (2)	0.98170 (17)	0.58884 (10)	0.0528 (4)	
O2	0.2367 (2)	0.33480 (17)	0.62755 (10)	0.0467 (3)	
H2O	0.232 (3)	0.227 (2)	0.6153 (17)	0.056*	
C1	0.2381 (3)	1.0221 (2)	0.38083 (14)	0.0390 (4)	
H1A	0.2107	1.1106	0.4190	0.047*	
C2	0.2633 (3)	1.0443 (2)	0.27863 (15)	0.0419 (4)	
C3	0.3060 (3)	0.9139 (3)	0.22129 (14)	0.0432 (4)	
C4	0.3205 (3)	0.7606 (3)	0.26707 (15)	0.0459 (4)	
H4A	0.3489	0.6729	0.2288	0.055*	
C5	0.2930 (3)	0.7374 (2)	0.36929 (14)	0.0410 (4)	
H5A	0.3012	0.6332	0.3992	0.049*	
C6	0.2531 (3)	0.8688 (2)	0.42820 (13)	0.0344 (3)	
C7	0.2250 (3)	0.8537 (2)	0.53956 (14)	0.0363 (4)	
C8	0.2227 (3)	0.6882 (2)	0.58971 (13)	0.0374 (4)	
H8A	0.2293	0.5889	0.5503	0.045*	
C9	0.2112 (3)	0.6809 (2)	0.69169 (14)	0.0386 (4)	
H9A	0.1997	0.7842	0.7240	0.046*	
C10	0.2133 (3)	0.5400 (2)	0.76114 (13)	0.0368 (4)	
C11	0.2267 (3)	0.3713 (2)	0.73036 (13)	0.0360 (4)	
C12	0.2295 (3)	0.2466 (3)	0.80426 (15)	0.0465 (4)	
H12A	0.2381	0.1349	0.7833	0.056*	
C13	0.2196 (4)	0.2864 (3)	0.90771 (16)	0.0578 (5)	
H13A	0.2220	0.2019	0.9565	0.069*	
C14	0.2061 (4)	0.4516 (3)	0.93985 (16)	0.0593 (6)	
H14A	0.1996	0.4787	1.0100	0.071*	
C15	0.2024 (3)	0.5748 (3)	0.86740 (15)	0.0506 (5)	
H15A	0.1923	0.6853	0.8894	0.061*	

### Atomic displacement parameters (Å^2^)

**Table:**

	*U*^11^	*U*^22^	*U*^33^	*U*^12^	*U*^13^	*U*^23^
Cl1	0.1015 (5)	0.0632 (4)	0.0687 (4)	0.0485 (3)	0.0365 (3)	0.0383 (3)
Cl2	0.0983 (5)	0.0790 (4)	0.0432 (3)	0.0402 (4)	0.0328 (3)	0.0210 (3)
O1	0.0915 (11)	0.0354 (7)	0.0465 (7)	0.0342 (7)	0.0276 (7)	0.0082 (6)
O2	0.0787 (9)	0.0325 (6)	0.0424 (7)	0.0290 (7)	0.0265 (7)	0.0083 (5)
C1	0.0470 (10)	0.0319 (8)	0.0425 (9)	0.0168 (7)	0.0144 (8)	0.0071 (7)
C2	0.0458 (10)	0.0374 (9)	0.0463 (10)	0.0174 (8)	0.0124 (8)	0.0157 (8)
C3	0.0481 (10)	0.0473 (10)	0.0360 (9)	0.0161 (9)	0.0139 (8)	0.0095 (8)
C4	0.0577 (11)	0.0426 (10)	0.0434 (10)	0.0216 (9)	0.0186 (9)	0.0037 (8)
C5	0.0536 (11)	0.0330 (8)	0.0415 (9)	0.0189 (8)	0.0153 (8)	0.0072 (7)
C6	0.0388 (9)	0.0281 (8)	0.0370 (8)	0.0121 (7)	0.0098 (7)	0.0048 (6)
C7	0.0440 (9)	0.0277 (8)	0.0390 (9)	0.0139 (7)	0.0118 (7)	0.0038 (7)
C8	0.0511 (10)	0.0261 (8)	0.0384 (9)	0.0158 (7)	0.0142 (8)	0.0037 (7)
C9	0.0514 (10)	0.0286 (8)	0.0380 (9)	0.0162 (7)	0.0123 (8)	0.0010 (7)
C10	0.0446 (9)	0.0329 (8)	0.0338 (8)	0.0143 (7)	0.0102 (7)	0.0039 (7)
C11	0.0423 (9)	0.0323 (8)	0.0359 (8)	0.0145 (7)	0.0121 (7)	0.0050 (7)
C12	0.0612 (12)	0.0397 (9)	0.0470 (10)	0.0247 (9)	0.0177 (9)	0.0135 (8)
C13	0.0765 (14)	0.0579 (12)	0.0472 (11)	0.0302 (11)	0.0180 (10)	0.0249 (10)
C14	0.0867 (16)	0.0629 (13)	0.0332 (9)	0.0297 (12)	0.0182 (10)	0.0101 (9)
C15	0.0771 (14)	0.0440 (10)	0.0370 (9)	0.0270 (10)	0.0180 (9)	0.0025 (8)

### Geometric parameters (Å, °)

**Table:**

Cl1—C2	1.7294 (17)	C7—C8	1.467 (2)
Cl2—C3	1.7241 (18)	C8—C9	1.330 (2)
O1—C7	1.226 (2)	C8—H8A	0.9300
O2—C11	1.358 (2)	C9—C10	1.448 (2)
O2—H2O	0.842 (16)	C9—H9A	0.9300
C1—C2	1.372 (2)	C10—C15	1.401 (2)
C1—C6	1.392 (2)	C10—C11	1.404 (2)
C1—H1A	0.9300	C11—C12	1.390 (2)
C2—C3	1.388 (3)	C12—C13	1.371 (3)
C3—C4	1.382 (3)	C12—H12A	0.9300
C4—C5	1.378 (2)	C13—C14	1.382 (3)
C4—H4A	0.9300	C13—H13A	0.9300
C5—C6	1.393 (2)	C14—C15	1.369 (3)
C5—H5A	0.9300	C14—H14A	0.9300
C6—C7	1.489 (2)	C15—H15A	0.9300
			
C11—O2—H2O	110.9 (15)	C9—C8—H8A	120.1
C2—C1—C6	120.90 (16)	C7—C8—H8A	120.1
C2—C1—H1A	119.6	C8—C9—C10	131.19 (15)
C6—C1—H1A	119.6	C8—C9—H9A	114.4
C1—C2—C3	120.06 (16)	C10—C9—H9A	114.4
C1—C2—Cl1	119.25 (14)	C15—C10—C11	117.39 (16)
C3—C2—Cl1	120.68 (14)	C15—C10—C9	117.66 (15)
C4—C3—C2	119.71 (16)	C11—C10—C9	124.96 (15)
C4—C3—Cl2	119.09 (14)	O2—C11—C12	121.68 (15)
C2—C3—Cl2	121.19 (14)	O2—C11—C10	118.26 (14)
C5—C4—C3	120.17 (16)	C12—C11—C10	120.06 (15)
C5—C4—H4A	119.9	C13—C12—C11	120.70 (17)
C3—C4—H4A	119.9	C13—C12—H12A	119.7
C4—C5—C6	120.58 (16)	C11—C12—H12A	119.7
C4—C5—H5A	119.7	C12—C13—C14	120.29 (18)
C6—C5—H5A	119.7	C12—C13—H13A	119.9
C1—C6—C5	118.56 (15)	C14—C13—H13A	119.9
C1—C6—C7	118.09 (14)	C15—C14—C13	119.36 (18)
C5—C6—C7	123.35 (14)	C15—C14—H14A	120.3
O1—C7—C8	120.65 (15)	C13—C14—H14A	120.3
O1—C7—C6	119.13 (14)	C14—C15—C10	122.21 (18)
C8—C7—C6	120.22 (14)	C14—C15—H15A	118.9
C9—C8—C7	119.71 (15)	C10—C15—H15A	118.9
			
C6—C1—C2—C3	−0.6 (3)	O1—C7—C8—C9	−3.9 (3)
C6—C1—C2—Cl1	178.20 (14)	C6—C7—C8—C9	175.47 (17)
C1—C2—C3—C4	0.8 (3)	C7—C8—C9—C10	−177.56 (18)
Cl1—C2—C3—C4	−177.92 (15)	C8—C9—C10—C15	178.7 (2)
C1—C2—C3—Cl2	−178.34 (14)	C8—C9—C10—C11	−0.9 (3)
Cl1—C2—C3—Cl2	2.9 (2)	C15—C10—C11—O2	179.76 (17)
C2—C3—C4—C5	−0.1 (3)	C9—C10—C11—O2	−0.6 (3)
Cl2—C3—C4—C5	179.07 (15)	C15—C10—C11—C12	−0.2 (3)
C3—C4—C5—C6	−0.9 (3)	C9—C10—C11—C12	179.39 (18)
C2—C1—C6—C5	−0.4 (3)	O2—C11—C12—C13	179.89 (18)
C2—C1—C6—C7	179.74 (17)	C10—C11—C12—C13	−0.1 (3)
C4—C5—C6—C1	1.1 (3)	C11—C12—C13—C14	0.2 (3)
C4—C5—C6—C7	−179.04 (17)	C12—C13—C14—C15	0.1 (4)
C1—C6—C7—O1	−5.7 (3)	C13—C14—C15—C10	−0.5 (4)
C5—C6—C7—O1	174.48 (18)	C11—C10—C15—C14	0.5 (3)
C1—C6—C7—C8	174.96 (16)	C9—C10—C15—C14	−179.1 (2)
C5—C6—C7—C8	−4.9 (3)		

### Hydrogen-bond geometry (Å, °)

**Table:**

*D*—H···*A*	*D*—H	H···*A*	*D*···*A*	*D*—H···*A*
O2—H2O···O1^i^	0.84 (2)	1.88 (2)	2.7168 (17)	176 (2)

Symmetry codes: (i) *x*, *y*−1, *z*.
